# Quality Differences in Frozen Mackerel According to Thawing Method: Potential Classification via Hyperspectral Imaging

**DOI:** 10.3390/foods13244005

**Published:** 2024-12-11

**Authors:** Seul-Ki Park, Jeong-Seok Cho, Dong-Hoon Won, Sang Seop Kim, Jeong-Ho Lim, Jeong Hee Choi, Dae-Yong Yun, Kee-Jai Park, Gyuseok Lee

**Affiliations:** 1 Smart Food Manufacturing Project Group, Korea Food Research Institute, Wanju 55365, Republic of Korea; skpark@kfri.re.kr (S.-K.P.); jscho@kfri.re.kr (J.-S.C.); jhlim@kfri.re.kr (J.-H.L.); choijh@kfri.re.kr (J.H.C.); jake@kfri.re.kr (K.-J.P.); 2 Food Safety and Distribution Research Group, Korea Food Research Institute, Wanju 55365, Republic of Korea; ehdgns3820@naver.com (D.-H.W.); sangseop.k@kfri.re.kr (S.S.K.); ydy0401@kfri.re.kr (D.-Y.Y.)

**Keywords:** frozen mackerel, thawing methods, hyperspectral imaging (HSI), food quality, spectroscopic analysis, seafood storage

## Abstract

Seafood quality preservation remains a critical focus in the food industry, particularly as the freeze–thaw process significantly impacts the freshness and safety of aquatic products. This study investigated quality changes in frozen mackerel subjected to two thawing methods, room temperature (RT) and running water (WT), and assessed the potential of hyperspectral imaging (HSI) for classifying these methods. After thawing, mackerel samples were stored at 5 °C for 21 days, with physicochemical, textural, and spectroscopic analyses tracking quality changes and supporting the development of a spectroscopic classification model. Compared with the WT method, the RT method delayed changes in key quality indicators, including pH, total volatile basic nitrogen (TVB-N), and total viable count (TVC), by 1–2 days, suggesting it may better preserve initial quality. Texture profile analysis showed similar trends, supporting the benefit of RT in maintaining quality. A major focus was on using HSI to assess quality and classify thawing methods. HSI achieved high classification accuracy (R_c_^2^ = 0.9547) in distinguishing thawing methods up to three days post-thaw, with 1100, 1200, and 1400 nm wavelengths identified as key spectral markers. The HIS’s ability to detect differences between thawing methods, even when conventional analyses showed minimal variation, highlights its potential as a powerful tool for quality assessment and process control in the seafood industry, enabling detection of subtle quality changes that traditional methods may miss.

## 1. Introduction

Food preservation has been a critical concern since the early days of agricultural societies [1,2]. Modern preservation methods primarily rely on refrigeration and freezing to extend shelf life and maintain food quality [3]. However, quality deterioration and value loss due to freeze–thaw processes continue to present significant challenges in food science [4]. Previous studies have extensively investigated the effects of freezing and thawing on food quality. For instance, Kaale et al. [5] reported that superchilling technology can effectively extend shelf life while maintaining food quality. Zhang et al. [6] demonstrated that multiple freeze–thaw cycles significantly affect moisture migration and cause microstructure damage in muscle tissues, leading to protein structure changes. Furthermore, Liu et al. [7] revealed that different storage temperatures (−3 °C and 0 °C) significantly influence biochemical and physical changes in fish fillets. Aquatic products, particularly fish, require special attention because of their high moisture content and the instability of their proteins, lipids, and tissue structures [8]. The perishable nature of seafood and the potential food safety risks associated with it complicate the maintenance of quality, safety, and storage stability across production, processing, storage, and distribution stages [9]. Lower-value aquatic products are typically distributed and sold having been either refrigerated shortly after harvest or frozen. Freezing is often preferred due to the high costs associated with maintaining the cold chain for refrigerated products. Advancements in freezing technology have extended the shelf life of mackerel [10], making the choice of thawing method a crucial factor in preserving product quality [11]. Recent studies have shown that different thawing conditions can significantly affect the final product quality. Wang et al. [12] found that various thawing methods significantly influence physicochemical properties and protein oxidation in muscle tissues. Their research showed that water-immersion thawing resulted in higher protein oxidation compared to air thawing and refrigeration thawing methods. Common industrial thawing methods include air thawing, underwater thawing, low-temperature thawing, ultrasonic thawing, and microwave thawing [13]. However, these methods can damage fish muscle tissues and skin, leading to quality degradation. Despite this, definitive methods to accurately quantify quality deterioration are missing. Conventional methods for assessing post-thaw freshness in seafood, such as pH measurement, viable cell counts, trimethylamine (TMA) analysis, total volatile basic nitrogen (TVB-N) levels, and texture analysis, are often destructive, time consuming, and limited in their ability to represent the overall quality of the sample [14].

To overcome these limitations, nondestructive analysis techniques such as hyperspectral imaging (HSI) have become increasingly popular [15]. HSI has emerged as an effective tool for assessing meat quality and freshness, including that of frozen and thawed products. Studies have shown that HSI can accurately distinguish between fresh, frozen, and frozen–thawed meat samples [16]. Recent research has even explored the use of HSI for evaluating frozen meat quality without the need for thawing. Xie et al. [17] demonstrated that HSI could assess various quality attributes of frozen pork, such as color, cooking loss, and drip loss, without requiring the samples to be thawed. This capability makes HSI a rapid, reliable, and nondestructive method for evaluating meat quality and freshness, particularly in frozen and thawed products. Its capability to provide spatially distributed spectral information also makes it a valuable tool for real-time monitoring and quality control in the meat industry [18].

This study presents a comprehensive approach by combining quality assessment methods with modern intervention technologies, addressing current challenges in the food industry. As Chen et al. [19] highlighted, the integration of various intervention technologies is crucial for ensuring food safety and quality in modern food processing. This study aimed to analyze the physicochemical quality changes induced by thawing in frozen mackerel and to assess thawing-induced quality degradation using hyperspectral imaging in the shortwave infrared range (HSI-SWIR). This study explored the potential of classifying mackerel based on the thawing method used. Although conventional physicochemical analyses may not differentiate between the two thawing methods, this study proposes that HSI can effectively classify quality changes according to the thawing method. This classification approach has potential industrial applications and could serve as a foundation for developing new technologies to measure thawing-related damage in conjunction with future advancements in thawing techniques. Furthermore, this approach not only advances our understanding of thawing-induced quality changes but also provides practical insights for industry application. The findings align with current trends in food safety and quality control technologies, as discussed by Chen et al. [19], potentially contributing to the development of more effective quality control processes in the seafood industry.

## 2. Materials and Methods

### 2.1. Sample Preparation and Thawing Conditions

The mackerel (*Scomber japonicus*) samples used in this study were sourced from the Cooperative Fishery Market in Busan, Republic of Korea. These samples were part of an initial batch distributed directly from fishing vessels, minimizing the time between catch and experimentation to ensure maximum freshness. Caught in October 2023, the mackerel had an average total length of 31.2 ± 1.5 cm and a mean weight of 361.5 ± 12.5 g. Samples were transported to the laboratory under temperature-controlled conditions (5 ± 0.5 °C) and used immediately to maintain consistent freshness among samples. For the frozen samples, mackerel was rapidly frozen to −20.0 ± 1.0 °C upon arrival, with core temperatures monitored using a probe thermometer. A 48 h freezing period was implemented to ensure complete freezing of the samples. Freezing was considered complete once the center of each sample reached −20.0 °C. Two thawing protocols were then employed: (a) room-temperature thawing (RT) at 25 ± 1.0 °C in a controlled laboratory setting, and (b) running water thawing (WT) in a water bath with circulating water at 15 ± 1.0 °C. Probe thermometers monitored core temperatures until they reached 4–5 °C. Samples were thawed either at room temperature for 6 h or under running water for 2 h until completion. Post-thaw, samples were stored at 5 ± 0.5 °C in the incubator (TH-TG, JEIO-Tech Co., Ltd., Seoul, Republic of Korea) and analyzed at intervals of 1, 2, 3, 5, 8, 14, and 21 days. Day 0 measurements were obtained from fresh samples prior to freezing. For each time point and condition, five mackerel samples were randomly selected for physicochemical and microbiological analysis. The entire muscle tissue from each sample was collected and homogenized (Ultra-Turrax T25, IKA Works, Inc., Wilmington, NC, USA) individually for total viable count (TVC), pH, titratable acidity, and total volatile basic nitrogen (TVB-N) measurements. All analyses were performed in triplicate.

### 2.2. Total Viable Cell Counts

The total viable cell counts (TVC) during mackerel storage were determined following the AOAC [20] method. Whole mackerel samples were homogenized at 10,000× *g*, and 25 g subsamples were mixed with phosphate-buffered saline at a 1:9 (*w*/*v*) ratio. The mixture was homogenized for 2 min using a stomacher (BagMixer 400 CC, Interscience, Ile-de-France, France). Serial dilutions were prepared using phosphate-buffered saline, and 1 mL of the appropriate dilutions was plated on plate count agar (Oxoid, UK). The plates were incubated at 30 ± 1 °C for 48 ± 2 h, after which the colonies were counted and converted to log CFU/g by multiplying by the dilution factor.

### 2.3. pH and Titratable Acidity

Whole mackerel samples were homogenized at 10,000× *g*, and 10 g of homogenate was mixed with nine times the volume of distilled water. This mixture was filtered using a Whatman No. 4 filter paper (Advantec, Tokyo, Japan), yielding 10 mL of filtrate for pH measurement. The pH was measured at room temperature (22 ± 1 °C) using a calibrated pH meter (Model A111, Thermo Scientific, Waltham, MA, USA). Titratable acidity (TA) was determined by titrating the filtrate with a standardized 0.1 N sodium hydroxide (NaOH) solution (Daejung Chmicals & Metals, Siheung, Republic of Korea) to an endpoint of pH 8.2. The volume of NaOH consumed was recorded, and the TA, expressed as percent lactic acid equivalent, was calculated using Equation (1).

(1) $$Titratable\;Acidity\;\left(\%\;lactic\;acid\right)=\frac{V_{NaOH}\times N_{NaOH}\times 0.090\times 100}{V_{Sample}}$$

*V*_NaOH_: volume of NaOH used (mL);
*N*_NaOH_: normality of the NaOH solution (0.1 N);
0.090: the milliequivalent factor for lactic acid;
*V*_sample_: volume of the sample (mL).

### 2.4. Total Volatile Basic Nitrogen (TVB-N)

TVB-N content was measured using a microdiffusion method described by Conway and Byrne [21], with some modifications performed according to the AOAC Official Method 971.14 [22]. A 1 mL aliquot of the supernatant, prepared as described in Section 2.3, was pipetted into the outer chamber of the Conway microdiffusion unit, whereas 1 mL of 0.01 N sulfuric acid (H_2_SO_4_) was added to the inner chamber. A saturated potassium carbonate (K_2_CO_3_) solution (1 mL) was added to the outer chamber to avoid contact with the test solution. The unit was sealed with a glass lid and clipped to ensure it was airtight. The contents were gently mixed by rotating the unit. The sealed Conway unit was then incubated at 25 ± 0.5 °C for 60 min to allow the volatile bases to fully diffuse. After incubation, the unit was carefully opened to avoid disturbing the contents. One drop (approximately 1 μL) of Brunswick indicator (a methyl red–methylene blue indicator) was added to the inner chamber. The contents of the inner chamber were then titrated with a standardized 0.01 N sodium hydroxide (NaOH) solution until the color shifted from red to blue-gray.

(2) $$Total\;volatile\;basic\;nitrogen\;(mg/100\;g)=\frac{\left(a-b\right)\times f\times 0.14\times d\times 100}{W}$$

*a*: volume (mL) of 0.01 N NaOH used for titrating the sample;
*b*: volume (mL) of 0.01 N NaOH used for titrating the blank;
*f*: factor of 0.01 N NaOH;
0.14: the milliequivalent of nitrogen;
*d*: dilution factor;
*w*: weight of the sample (g).

### 2.5. Texture Profile Analysis

**Figure 1:**
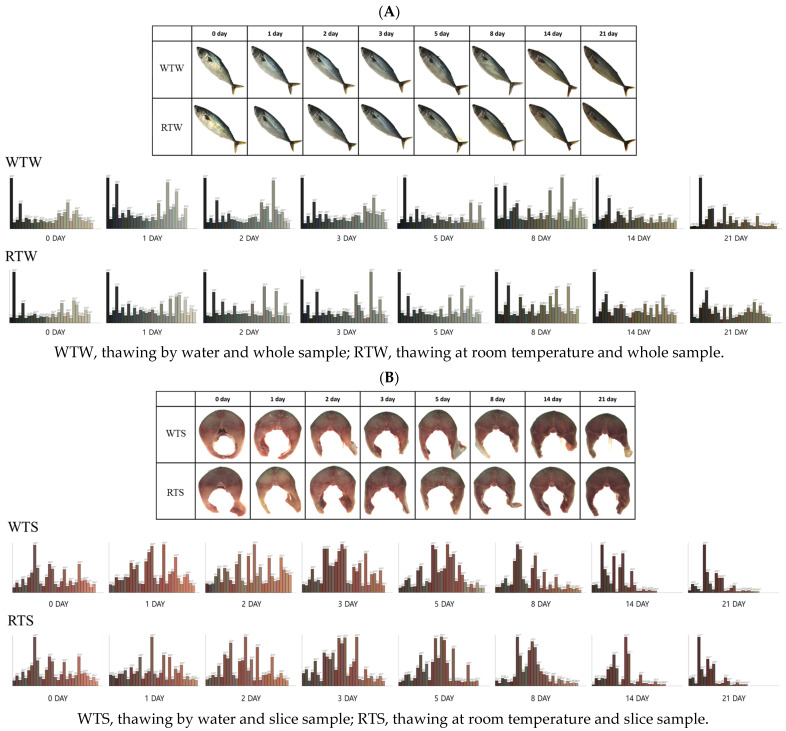
Physical state and color changes in whole (**A**) and sliced (**B**) mackerel samples during storage according to the thawing method used.

Texture profile analysis (TPA) was conducted using a TA.XTplus Texture Analyzer (Stable Micro Systems Ltd., Surrey, UK) with a 5 kg load cell following a modified method from Cheng et al. [23]. After storage at each temperature, mackerel fillets were carefully cut into uniform cuboids measuring 1.5 cm × 1.5 cm × 2.0 cm (length × width × height) using a sharp knife. The samples were obtained from the dorsal muscle region, as this area provides sufficient muscle mass for consistent cube-shaped samples (Figure 1B). The samples were then allowed to equilibrate to room temperature (22 ± 1 °C) for 30 min before analysis. A 100-diameter compression plate probe (P/100) was used for the double compression test. The TPA was conducted under the following conditions: pre-test speed of 1.0 mm/s, test speed of 1.0 mm/s, post-test speed of 1.0 mm/s, target strain of 70%, trigger force of 5 g, and a 3 s time interval between the two compression cycles. The probe compressed each sample to 70% of its original height. Six textural parameters were calculated from the resulting force–time curves using Texture Exponent software (version 6.1.16.0, Stable Micro Systems, Godalming, UK): hardness (N), defined as the peak force during the first compression cycle; cohesiveness (dimensionless), the ratio of the positive force area during the second compression to that during the first compression; springiness (mm), the height recovered by the sample between the end of the first compression and the start of the second compression; gumminess (N), calculated as hardness × cohesiveness; chewiness (N·mm), calculated as gumminess × springiness; and resilience (dimensionless), the ratio of upstroke energy to downstroke energy during the first compression. Ten replicate measurements were performed for each treatment, with samples taken from different parts of the fillets to account for potential variations in muscle structure. Results are expressed as mean ± standard deviation.

### 2.6. Color Analysis

Color analysis of thawed mackerel samples was conducted using a method adapted from Chaijan et al. [23] and Sánchez-Alonso et al. [24]. After storage at each temperature, samples were divided into two groups based on the preparation method: whole fish and sliced portions. For the sliced group, uniform slices 2.0 cm thick were carefully cut from the dorsal muscle, parallel to the fish’s long axis, using a sharp knife to maintain consistency while observing the color change in the internal muscles. Color measurements were performed using a Konica Minolta CM-700d Spectrophotometer (Konica Minolta Sensing Inc., Osaka, Japan) equipped with an 8 mm diameter measuring aperture, D65 illuminant, and a 10° standard observer. The instrument was calibrated using a white calibration plate and a zero-calibration box before each series of measurements. To derive RGB values from the spectrophotometric data, a series of colorimetric transformations was applied. This process involved converting Lab* coordinates to XYZ tristimulus values using standardized conversion matrices, followed by transforming XYZ values to RGB coordinates using a color-space-specific transformation matrix (e.g., sRGB). Finally, gamma correction was applied to produce accurate RGB values, accounting for the nonlinear responses of display devices. The extracted RGB color codes were visualized to facilitate data interpretation. Measured RGB values were represented as actual colors, and variations in these RGB colors were depicted using bar graphs. This comprehensive analytical approach was applied to both whole fish specimens and sliced samples, aiding in the visual assessment of color distribution.

### 2.7. Hyperspectral Image Acquisition

Hyperspectral image acquisition was conducted using a custom-built shortwave infrared HSI (SWIR-HSI) system. The SWIR-HSI setup included an SWIR camera (PA320F300TCL, OZRAY, Gwangmyeong, Republic of Korea), a spectrograph (N17E, Specim, Oulu, Finland), a lens (HS1214V-SW, uTRON, Tokyo, Japan), a vision dome light (VTDL550*240, Vision Technology, Cheonan-si, Republic of Korea) equipped with six 150 W halogen lamps, and a sample linear stage (FBL80E1400, FUYU, Chengdu, China). The system operated within a spectral range of 900–1700 nm and had an image resolution of 320 × 315 pixels. Images were captured using a line-scan (push-broom) method, where a step motor moved the sample at a constant speed of 275 mm/s to match the frame rate of the camera (10 fps). For sample preparation, thawed mackerel specimens were placed on a non-reflective black background, with each mackerel positioned consistently, left eye facing upward. A total of 100 mackerels (50 per day for each thawing group) were analyzed, with measurements taken eight times over a 21-day storage period to monitor changes over time. Image processing and spectral data extraction were performed using the Region of Interest function in the PerClass software (version 3.0.7, PerClass BV, Delft, The Netherlands), allowing for the isolation and analysis of specific areas within each hyperspectral image. The HSI data collection method used here was adapted from the protocols of Cheng et al. [25] and Cho et al. [26], with modifications to accommodate the specific needs of mackerel analysis.

### 2.8. Model Development and Performance Evaluation

An internal quality discriminant calibration model using HSI reflectance spectra was developed using partial least squares discriminant analysis (PLS-DA) [27,28]. PLS-DA is a supervised classification method that uses nondestructively measured spectral data as predictor variables and quality parameters associated with different thawing methods as response variables, allowing samples to be classified into predefined categories. The high-dimensional spectral data were reduced to a set of latent variables that maximized covariance between the predictor variables (spectral data) and response categories (thawing methods).

The calibration model was developed using Unscrambler X software (version 10.2; CAMO Software AS, Oslo, Norway). Prior to model development, spectral preprocessing techniques such as multiplicative scatter correction and the standard normal variate method were applied to minimize light scattering effects and baseline variations, thus enhancing model accuracy. The performance of the calibration model was evaluated using metrics such as the coefficient of determination for calibration (R_c_^2^) and the root mean square error of calibration (RMSEC). Classification accuracy was calculated as the average percentage of correctly classified RT and WT test samples in the final classification model. A total of 400 hyperspectral data points from storage days 0 to 3 were used to build the classification model, with 70% of the data allocated for calibration and the remaining 30% used to assess discriminant accuracy.

### 2.9. Statistical Analysis

All experiments included at least five replicates. Statistical analyses were performed using SPSS software (version 23.0; IBM Corp., Armonk, NY, USA). One-way analysis of variance was used to assess significant differences among samples (*p* < 0.05). Duncan’s multiple range test (*p* < 0.05) was used for post hoc analysis to identify specific group differences when applicable.

## 3. Results and Discussion

### 3.1. Microbiological and Physicochemical Changes

The results of quality indicators and total bacterial counts measured over 21 days of storage at 5 °C after thawing frozen mackerel using two methods are shown in Table 1. Statistically significant differences (*p* < 0.05) were observed in pH, TVB-N, and TVC depending on the thawing method, although no significant differences in TA were found. Fresh fish generally have a pH range of 5.5 to 6.5. After death, glycolysis produces lactic acid, which lowers the pH to approximately 5.6–5.8, particularly in red fish species [29]. Over time, enzymatic and microbial activity in fish causes protein decomposition, raising the pH and producing compounds such as amino acids, ammonia, peptides, and amines [30]. Despite the use of fresh mackerel in this experiment, the initial pH after thawing and at the start of storage was 6.38 ± 0.07. RT, representing a slower thawing process, showed the lowest pH, with no significant change until around day 5 of storage. In contrast, WT, a faster thawing method, showed a significant pH increase starting from storage day 1. Statistical differences in pH were noted from days 0 and 1 to days 5, 8, and 14–21, effectively categorizing the process of post-thaw freshness loss and spoilage into four distinct freshness stages. This trend was also observed in other physicochemical and microbiological indicators, which displayed statistically significant changes. The WT group showed the lowest pH value only on day 0, while the RT group maintained similar pH values on days 0–5. Both groups exhibited similar pH changes from day 8 onward. These results align with those of Park et al. [31], who reported that the pH increased during fish storage. However, our study demonstrated significant differences depending on the thawing method used. For TVB-N, the RT group showed no significant changes until day 2, while the WT group remained stable only until day 1, with changes beginning on day 2. By day 21, both groups showed similar TVB-N values, though the RT method slightly delayed the TVB-N increase during the early storage period (up to day 3) post-thaw. In terms of TVC, results were statistically lower on day 1 of storage compared to pre-storage levels, likely due to a reduction in the microorganism levels during freezing [32]. Microbial growth was slower in the RT group, with TVC increasing from day 5 of storage, whereas the WT group showed significant TVC increases starting on day 2 of storage. Previous studies have reported that muscle tissue damage from freezing and thawing accelerates spoilage during storage [33]. We hypothesized that the WT group, having undergone faster thawing, experienced greater muscle tissue damage than the RT group, creating a more favorable environment for microbial growth. Analysis of TVC, pH, TA, and TVB-N across thawing methods indicated that the RT group’s method extended the freshness period by approximately 1–2 days, delaying changes in all parameters, except for TA. These differences may reflect the varying degrees of muscle tissue damage caused by the different thawing methods. The present study aimed to distinguish changes based on thawing method by analyzing physicochemical changes and hyperspectral data.

### 3.2. Texture Profile Analysis by Thawing Method

Table 2 shows the TPA results for fresh mackerel affected by different thawing methods over the storage period. Statistically significant changes were observed in hardness, gumminess, and chewiness, whereas no significant differences were noted in cohesiveness, springiness, and resilience. This section thus focuses on hardness, gumminess, and chewiness. These three texture parameters followed a similar pattern: a significant decrease from day 0 to day 3 of storage, followed by an increase from day 5 to day 21. The initial hardness of the mackerel sample on day 0 was 1311.71 g. On day 1 of storage, hardness values were 1062 g and 1081 g for the WT and RT groups, respectively. The RT group maintained a hardness of 1011 g until day 2 of storage, whereas the WT group had a lower hardness of approximately 850 g from day 2 to day 3. The hardness of the RT group dropped to 647 g by day 3. The RT group maintained hardness values above 1000 g until day 2, whereas the hardness of the WT group fell below 1000 g after day 1. Both groups showed their lowest hardness on day 3 of storage (*p* < 0.05). Liang et al. [34] reported that, in mackerel muscle tissue stored at 5 °C, the stained muscle fiber area decreased rapidly from 82.88% on day 0 to 65.78% on day 1, 62.39% on day 3, and 58.92% on day 5, indicating significant reductions in muscle fiber density during early storage.

In this study, the initial decrease in hardness (days 1–3) was likely due to rapid muscle contraction causing gaps and tissue softening. After day 5, increased viscosity from complete muscle tissue deterioration may have contributed to the observed hardness increase. This trend was also seen in gumminess and chewiness. Gumminess in the WT group decreased sharply from day 2 to day 3, with values of 215 and 204, respectively. Interestingly, the RT group maintained a gumminess value of 270 or higher until day 2 of storage. Although the reason is unclear, the gumminess of the RT group on day 3 was lower than that of the WT group, mirroring the hardness results. Chewiness followed similar trends, with the WT group showing values of 98.40 on day 1 and 64.62 on day 2, whereas the RT group showed values of 80.31 on day 1 and 83.66 on day 2. On day 3, the chewiness values were 64.98 for the WT group and 54.09 for the RT group. The lower hardness, gumminess, and chewiness values in the RT group on day 3 compared to those of the WT group are not fully understood. However, the texture profile changes in the RT group were delayed by approximately one day compared with those in the freshest samples (day 0), consistent with the observed physicochemical changes.

Textural changes in refrigerated fish are influenced by various factors, and numerous studies have investigated this phenomenon. Studies have demonstrated that fish frozen for long periods tend to develop a tougher texture and experience a decline in overall quality [35,36]. Sharifian et al. [37] observed that muscle fibers in grouper fillets stored in a refrigerator initially appeared homogeneous with a normal cross-sectional shape; however, after seven days, the extracellular space and fibers contracted, worsening over time and affecting texture. Similarly, Suárez et al. [38] found that sea bream muscle firmness was retained longer at 1 °C than at 4 °C. They also found that muscle collagen decomposition occurred more rapidly at 4 °C than at 1 °C, with faster collagen degradation occurring at 4 °C and contributing to firmness loss by affecting cross-linking. These studies highlight the many factors influencing textural changes in fish samples. The findings from this study suggest that texture change profiles vary due to the complex interactions between freezing, thawing, and storage conditions, among others. Compared to those in fresh samples (day 0), texture parameters such as hardness, gumminess, and chewiness exhibited distinct changes depending on the thawing method. Notably, the RT method appeared to delay these changes by about one day compared to the WT method. This delayed texture change obtained using the RT method suggests it may be preferable for maintaining the textural quality of frozen–thawed mackerel for a slightly longer period.

### 3.3. Investigation of RGB Color Change

The changes in the RGB values of the mackerel samples during storage, influenced by different thawing methods, are presented in Table 3 and Figure 1. To aid visual comprehension of the color analysis, Figure 1 includes representative photographs of the actual samples and bar graphs depicting the measured RGB values. Color analysis was conducted daily on whole specimens and cross-sections (slices) of the samples. All R, G, and B values gradually decreased as storage progressed, resulting in an overall darkening of the samples (Figure 1). Significant changes in RGB values were observed after 3 to 5 days of storage, with no statistically significant changes prior to this period (*p* < 0.05). The red (R) values of room-temperature-thawed and sliced samples (RTS) remained higher for approximately two days longer than those of water-thawed and sliced samples (WTS), after which both groups showed a declining trend. For whole samples, both the water-thawed whole-sample and room-temperature-thawed whole-sample groups exhibited a sharp decrease in R values starting from day 2 of storage. These findings align with those reported by Chaijan et al. [23], who observed a similar decrease in red values in mackerel during ice storage. The green (G) values for sliced samples ranged from 97.22 to 80.67, with RTS samples maintaining G values above 90 for up to 14 days. All samples exhibited a continuous decrease in G values, from a maximum of 133.08 to a minimum of 77.50. No significant differences in G values were observed between the WT and RT samples. In the blue (B) spectrum, RTS samples maintained values above 80 for up to 14 days of storage, whereas WTS samples maintained values above 80 for only 3 days. The whole-sample group showed a continuous decrease in B values from 114.98 to 62.40 throughout the storage period, with no specific trends based on the thawing method used. Traditionally, color analysis has focused on basic sensory changes; however, recent advancements have enabled the use of nondestructive techniques to assess food and seafood quality by using color data (RGB or L*, a*, b* values) as proxies for sensory attributes. For instance, Genç [39] utilized a feedforward artificial neural network model to predict storage time based on L*, a*, and b* values from 205 literature sources. Moreover, Khoshnoudi-Nia and Moosavi-Nasab [40] evaluated a multispectral imaging system to predict various freshness indicators in rainbow trout fillets. Such studies demonstrate the potential of color values and imaging data to assess seafood quality. In this study, RGB color codes were extracted, visualized, and measured to enhance our understanding of quality changes in mackerel during storage under different thawing conditions. This approach aims to provide insights into quality changes and support the development of nondestructive quality assessment methods for seafood.

### 3.4. Characteristics of Hyperspectral Reflectance Spectra

**Figure 2:**
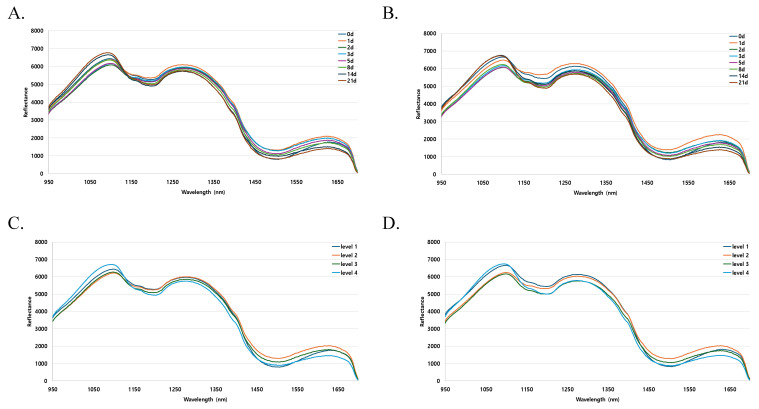
Average spectra of mackerel samples by thawing method obtained using hyperspectral imaging. (**A**) Room-temperature thawing (RT) over the full storage duration; (**B**) water thawing (WT) over the full storage duration; (**C**) RT grouped into 4 freshness levels; (**D**) WT grouped into 4 freshness levels.

Figure 2 presents the average reflectance spectra of the mackerel samples during storage, highlighting the effects of different thawing methods. Figure 2A,C show the mean spectra for RT samples, whereas Figure 2B,D show the spectra obtained for WT samples. Figure 2A,B illustrate spectral data by storage day, whereas Figure 2C,D categorize data into four freshness levels based on physicochemical analysis: level 1 (fresh) on day 0, level 2 (moderate freshness) for days 1–3 post-thawing, level 3 (early spoilage) for days 5–8, and level 4 (complete spoilage) for days 14–21. Spectroscopy, which relies on light absorption by molecules [41], allows for rapid, nondestructive assessment of chemical composition and bonding changes. Both the RT and WT groups showed similar overall spectral patterns; however, distinct spectral variations were observed on specific storage days, particularly at wavelengths of 1100, 1200, and 1600 nm (Figure 2A,B). For the RT samples, significant differences in the mean spectra appeared at 1100 and 1600 nm across storage days. For the WT samples, notable differences were observed at 1100, 1200, and 1600 nm. In the RT group, the spectra at 1100 nm clustered into groups by storage day (21 and 14 days, 0, 1, and 8 days, and 2, 3, and 5 days), while, at 1600 nm, each storage day was differentiated. The WT group showed similar clustering at 1100 nm, with additional separation at 1200 nm between days 0 and 1 and the other storage days. Additionally, for the WT, notably higher reflectance intensity was observed on day 1 than on other storage days in the 1600 nm region. When grouped into four freshness levels based on physicochemical analysis (Figure 2C,D), the RT samples showed differences in reflected intensity at specific wavelengths: 1100 nm (levels 1 and 4 vs. 2 and 3), 1200 nm (levels 1 and 2 vs. 3 and 4), and 1600 nm (level 2 vs. levels 1 and 3 vs. level 4). Cho et al. [26] reported similar findings, observing distinct differences in reflected intensity at 1200 nm in mean spectra of mackerel eyes and whole bodies during storage, achieving a prediction accuracy of 90.14% by correlating spectral data with TVB-N levels, further validating the potential of HSI for accurate freshness assessment. These spectral variations likely reflect freshness loss due to microbial and enzymatic activity [42,43] and chemical–physical changes in fish muscle. In summary, the mean spectral analysis in this study highlights the potential of HSI to interpret changes and detect tissue damage resulting from different thawing methods, differences that may not be easily distinguishable using conventional physicochemical analyses.

In conclusion, the mean spectral analysis in this study confirms that HSI is a powerful tool for detecting thawing-induced changes that are challenging to observe using traditional physicochemical methods. The observed spectral differences between RT and WT samples at specific wavelengths (1100, 1200, and 1600 nm) suggest that different thawing methods may induce unique chemical and structural changes in mackerel tissue. These spectral signatures could serve as nondestructive indicators of fish freshness and quality, offering deeper insights into the impact of thawing methods on seafood products.

### 3.5. Spectrum Classification According to the Thawing Method

This study aimed to classify changes in mackerel samples due to different thawing methods using spectroscopic data linked to physicochemical parameters. As shown in Figure 1, distinguishing storage duration visually is challenging. Table 4 shows that the PLS-DA model demonstrated varying performance in differentiating thawing methods across storage periods. For fresh samples on day 0, the model achieved an R_c_^2^ of 0.6734, an RMSEC of 0.4813, and a classification accuracy of 80%. According to the experimental protocol, day 0 samples consisted of fresh, unfrozen mackerel, resulting in significant model variation compared to thawed samples analyzed on subsequent days (days 1–3). On day 1, the performance of the model improved, reaching an R_c_^2^ of 0.9547, an RMSEC of 0.1064, and a classification accuracy of 100%. This high accuracy persisted on day 3, with an R_c_^2^ of 0.8168, an RMSEC of 0.2140, and 100% classification accuracy. On day 2, the model achieved an R_c_^2^ of 0.7683, an RMSEC of 0.2407, and a classification accuracy of 96.67%. These findings suggest that the accuracy of the PLS-DA model in distinguishing thawing methods improves with storage time, achieving optimal accuracy from day 1 onwards. However, beyond day 5 of storage, the accuracy of the model deteriorated significantly, likely due to biochemical degradation in the mackerel tissue, whose underlying mechanisms and metabolic changes require further investigation. Visual inspection alone proved insufficient for distinguishing samples by the thawing method. Although color analysis showed general trends of darkening and reduced saturation, it did not enable classification according to the thawing method for either whole or sliced samples (Figure 1). In contrast, PLS plots of the spectral data seem to enable classification according to the thawing method up to the third day of storage. These findings suggest that spectroscopic techniques can be used to monitor and classify changes due to thawing methods used for thawing seafood products. Tang et al. [44] established a linear relationship between spectral reflectance and the texture profile of crispy tilapia using VIS/NIR and SWIR spectroscopy. Similarly, Li et al. [45] conducted a study that classified minced salmon (*Salmo salar*) adulterated with *Oncorhynchus mykiss* (rainbow trout) and *Oncorhynchus keta* (chum salmon) in 10% (*w*/*w*) increments. Similar to these studies, which demonstrate the effectiveness of SWIR for food adulteration analysis, our research highlights the potential of using HSI to classify quality changes during storage based on thawing methods. While physicochemical analysis and texture profiling between the RT and WT groups revealed observable but subtle differences, these distinctions may have gone unnoticed without a direct comparison between the two thawing methods. However, by accumulating HSI data and utilizing machine learning, this study demonstrates the possibility of classifying samples based on thawing methods. This approach offers a more sensitive and objective means of assessing quality changes in fish products, potentially revealing insights that traditional analytical methods might miss.

### 3.6. Identification of Key Wavelengths Using PLS-DA Beta Coefficient Analysis

**Figure 3:**
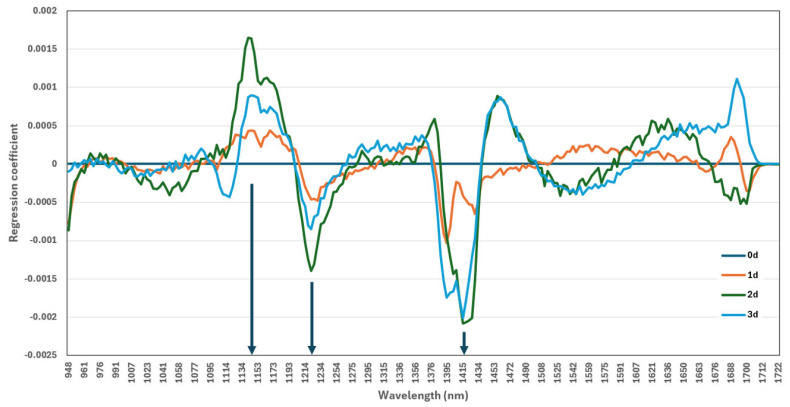
Beta coefficients of the PLS-DA model developed using raw hyperspectral data.

The beta coefficients derived from the PLS-DA model using full-wavelength spectra from the early storage period (0–3 days) are shown in Figure 3. As Cheng and Sun (2015) have noted, these coefficients help identify influential wavelengths in the PLS-DA model, facilitating the selection of key spectral components for classification. Analysis of the major peaks in regression coefficients identified significant wavelengths (1140, 1220, and 1400 nm) (Figure 3).

The 1100 nm wavelength in SWIR corresponds to the second overtone of C-H stretching vibrations, which are essential for detecting organic molecules in biological tissues [46]. The 1200 nm wavelength, similar to the second overtone of C-H stretching, is valuable for assessing the presence of lipids and fats in food products, providing insights into fat content and composition [47]. Additionally, the 1400 nm wavelength is critical for SWIR spectroscopy due to its association with the first overtone of O-H stretching, which is directly related to water content in tissues [48]. The sensitivity of this wavelength to water makes it essential for analyzing hydration levels in biological tissues.

Wilson et al. [46] noted that the SWIR range is particularly advantageous for non-invasive tissue analysis, since its deeper penetration compared to that of visible light allows for more thorough examination of tissue composition changes. In this study, the 1100 nm wavelength correlated with variations in lipid content within the tissue, whereas the changes at 1200 nm appear to reflect muscle oxygenation status, potentially indicating variations in tissue perfusion [49]. Furthermore, the 1400 nm wavelength was sensitive to water content changes, indicating tissue hydration levels or potential edema in stored samples [46]. The spectral peaks observed at 1100, 1200, and 1400 nm align with known molecular changes in food tissues, making these wavelengths promising indicators for quality classification.

## 4. Conclusions

This study aimed to assess the feasibility of differentiating frozen mackerel samples subjected to various thawing methods using physicochemical, physical, and spectroscopic data. Significant differences between RT and WT samples were observed in the physicochemical properties and TPA results, with additional distinctions evident in HSI spectral data. PLS-DA of the HSI data demonstrated high classification accuracy (maximum R_c_^2^ = 0.9547) from day 1 to day 3 of storage, identifying the wavelengths of 1100, 1200, and 1400 nm as the most influential. While future research is needed to determine the specific components associated with these wavelengths, this study successfully demonstrates the viability of using them as spectroscopic indicators for classifying quality changes in mackerel due to different thawing methods. Although additional investigation is required to clarify the causal relationships between these spectral regions and the changes in food components caused by different thawing methods, the findings of this study are highly valuable and significant. Moreover, this research holds substantial potential for practical applications, particularly in addressing the gap between consumer preference for refrigerated seafood and the industry’s practice of freezing and thawing products to extend shelf life and improve cost efficiency. The ability to differentiate thawing methods using HSI up to three days post-thawing is especially noteworthy, suggesting that the initial thawing process induces persistent changes in fish tissues that can be detected spectroscopically, even when not observable through conventional sensory or physicochemical analyses. This capability could prove valuable for quality control in the seafood industry, potentially enabling the identification of products that have undergone suboptimal thawing.

## Figures and Tables

**Table 1:** Quality indicators and microbiological analysis of thawed mackerel samples by storage day.

Traits	Thawing Condition	Storage Days (Day)							
		0	1	2	3	5	8	14	21
pH	WT *	6.38 ± 0.07 ^a^**	6.47 ± 0.11 ^ab^	6.49 ± 0.09 ^ab^	6.53 ± 0.03 ^ab^	6.55 ± 0.04 ^ab^	6.64 ± 0.07 ^b^	6.85 ± 0.22 ^c^	6.90 ± 0.29 ^c^
	RT *	6.38 ± 0.07 ^a^	6.42 ± 0.04 ^a^	6.38 ± 0.07 ^a^	6.45 ± 0.05 ^ab^	6.41 ± 0.08 ^a^	6.61 ± 0.05 ^b^	6.84 ± 0.18 ^c^	6.88 ± 0.29 ^c^
Titratable Acidity (%)	WT	1.79 ± 0.16 ^b^	1.74 ± 0.23 ^b^	1.76 ± 0.23 ^b^	1.78 ± 0.02 ^b^	1.67 ± 0.07 ^b^	1.62 ± 0.11 ^b^	1.31 ± 0.24 ^a^	1.32 ± 0.28 ^a^
	RT	1.79 ± 0.16 ^bc^	2.03 ± 0.07 ^d^	1.92 ± 0.07 ^cd^	1.87 ± 0.12 ^cd^	1.81 ± 0.08 ^bcd^	1.60 ± 0.15 ^ab^	1.38 ± 0.14 ^a^	1.52 ± 0.33 ^a^
TVB-N (mg/100 g)	WT	12.27 ± 1.54 ^a^	11.78 ± 1.60 ^a^	12.98 ± 0.70 ^ab^	16.27 ± 0.77 ^bc^	18.52 ± 1.17 ^cd^	20.20 ± 1.25 ^d^	44.33 ± 2.91 ^e^	64.81 ± 6.90 ^f^
	RT	12.27 ± 1.54 ^a^	11.78 ± 0.77 ^a^	12.91 ± 1.17 ^a^	16.55 ± 1.17 ^b^	18.80 ± 0.77 ^bc^	19.92 ± 1.17 ^c^	43.21 ± 3.04 ^d^	69.30 ± 4.82 ^e^
Total Viable Cell Count (Log CFU/g)	WT	3.71 ± 0.30 ^d^	2.20 ± 0.15 ^a^	2.74 ± 0.30 ^b^	3.15 ± 0.22 ^bc^	3.56 ± 0.23 ^cd^	4.34 ± 0.46 ^e^	4.90 ± 0.52 ^f^	5.56 ± 0.21 ^g^
	RT	3.71 ± 0.30 ^b^	2.56 ± 0.70 ^a^	3.02 ± 0.34 ^a^	2.87 ± 0.19 ^a^	3.86 ± 0.42 ^bc^	4.30 ± 0.12 ^c^	5.24 ± 0.45 ^d^	5.20 ± 0.11 ^d^

* WT, water thawing; RT, thawing at room temperature. ** Means within a column with different superscripts are significantly different (*p* < 0.05) in the same thawing condition.

**Table 2:** Texture profile analysis of thawed mackerel samples by storage day.

Traits	Thawing Condition	Storage Days (Day)							
		0	1	2	3	5	8	14	21
Hardness	WT *	1311.71 ± 234.62 ^d^*	1062.78 ± 109.68 ^bc^	848.04 ± 131.55 ^a^	854.83 ± 191.14 ^a^	1012.76 ± 85.33 ^ab^	1040.85 ± 75.64 ^bc^	1184.86 ± 123.12 ^bcd^	1210.83 ± 69.11 ^cd^
	RT^*^	1311.71 ± 234.62 ^cd^	1081.12 ± 78.35 ^b^	1011.82 ± 175.00 ^b^	647.10 ± 238.67 ^a^	1036.57 ± 157.63 ^b^	1054.22 ± 156.20 ^b^	1156.11 ± 95.32 ^bc^	1480.51 ± 87.74 ^d^
Cohesiveness	WT	0.27 ± 0.01 ^bc^	0.27 ± 0.02 ^c^	0.24 ± 0.03 ^ab^	0.24 ± 0.02 ^a^	0.30 ± 0.03 ^d^	0.28 ± 0.02 ^cd^	0.28 ± 0.02 ^cd^	0.28 ± 0.02 ^cd^
	RT	0.27 ± 0.01 ^bc^	0.24 ± 0.01 ^a^	0.27 ± 0.01 ^cd^	0.25 ± 0.02 ^ab^	0.28 ± 0.02 ^cd^	0.28 ± 0.02 ^cd^	0.26 ± 0.01 ^bc^	0.29 ± 0.01 ^d^
Springiness	WT	0.29 ± 0.01 ^a^	0.34 ± 0.04 ^bc^	0.32 ± 0.01 ^ab^	0.31 ± 0.01 ^ab^	0.35 ± 0.03 ^cd^	0.36 ± 0.03 ^cd^	0.32 ± 0.01 ^ab^	0.38 ± 0.02 ^d^
	RT	0.29 ± 0.01^a^	0.32 ± 0.04 ^ab^	0.30 ± 0.02 ^a^	0.35 ± 0.03 ^bcd^	0.35 ± 0.02 ^bcd^	0.36 ± 0.02 ^cd^	0.34 ± 0.03 ^bc^	0.38 ± 0.04 ^d^
Gumminess	WT	345.76 ± 51.11 ^b^	293.28 ± 44.54 ^b^	215.06 ± 46.57 ^a^	204.78 ± 51.16 ^a^	313.23 ± 31.02 ^b^	293.14 ± 31.34 ^b^	329.50 ± 22.21 ^b^	328.65 ± 40.37 ^b^
	RT	345.76 ± 51.11 ^c^	240.17 ± 18.45 ^b^	273.05 ± 38.73 ^b^	160.23 ± 87.17 ^a^	281.68 ± 28.41 ^b^	289.48 ± 40.30 ^b^	298.77 ± 32.50 ^bc^	409.76 ± 31.82 ^d^
Chewiness	WT	100.59 ± 9.96 ^b^	98.40 ± 13.66 ^b^	64.62 ± 14.76 ^a^	64.98 ± 13.93 ^a^	112.70 ± 13.99 ^bc^	109.97 ± 19.34 ^bc^	110.24 ± 14.42 ^bc^	119.86 ± 9.19 ^c^
	RT	100.59 ± 9.96 ^bc^	80.31 ± 17.17 ^b^	83.66 ± 19.05 ^b^	54.09 ± 24.99 ^a^	100.01 ± 16.16 ^bc^	111.36 ± 29.65 ^c^	105.29 ± 16.37 ^bc^	158.10 ± 24.40 ^d^
Resilience	WT	0.10 ± 0.00 ^a^	0.09 ± 0.01 ^a^	0.10 ± 0.01 ^a^	0.10 ± 0.01 ^a^	0.09 ± 0.01 ^a^	0.09 ± 0.00 ^a^	0.09 ± 0.00 ^a^	0.09 ± 0.01 ^a^
	RT	0.10 ± 0.00 ^d^	0.09 ± 0.01 ^cd^	0.09 ± 0.00 ^bcd^	0.09 ± 0.01 ^ab^	0.09 ± 0.01 ^abcd^	0.09 ± 0.01 ^abc^	0.08 ± 0.01 ^a^	0.10 ± 0.01 ^d^

WT, thawing by water; RT, thawing at room temperature. * Means with different superscripts within each row indicate significant differences by Duncan’s multiple range test (*p* < 0.05).

**Table 3:** RGB value analysis of frozen mackerel samples based on the thawing method used and storage duration.

Traits	Thawing Condition	Storage Days (Day)							
		0	1	2	3	5	8	14	21
Red (R)	WTS *	139.39 ± 7.80 ^c^**	154.12 ± 5.13 ^d^	141.03 ± 12.69 ^c^	132.66 ± 10.05 ^bc^	123.43 ± 12.36 ^ab^	123.59 ± 5.95 ^ab^	125.25 ± 10.71 ^ab^	113.53 ± 7.08 ^a^
	RTS *	139.39 ± 7.80 ^cd^	126.97 ± 9.34 ^b^	142.13 ± 7.60 ^d^	132.45 ± 16.87 ^cd^	140.10 ± 13.50 ^cd^	126.94 ± 8.68 ^b^	131.26 ± 7.99 ^cd^	111.04 ± 5.05 ^a^
	WTW *	133.44 ± 10.73 ^c^	123.07 ± 6.65 ^bc^	112.53 ± 5.81 ^ab^	106.83 ± 8.01 ^a^	106.38 ± 5.73 ^a^	108.44 ± 4.17 ^a^	101.20 ± 5.17 ^a^	86.21 ± 4.13 ^a^
	RTW *	132.95 ± 10.88 ^c^	116.03 ± 4.55 ^b^	111.41 ± 7.13 ^ab^	103.05 ± 0.87 ^a^	110.33 ± 5.20 ^ab^	103.56 ± 3.38 ^a^	100.51 ± 2.33 ^a^	84.67 ± 2.84 ^a^
Green (G)	WTS	97.22 ± 7.62 ^bc^	101.59 ± 4.16 ^d^	99.51 ± 10.03 ^d^	93.49 ± 7.93 ^bc^	88.14 ± 7.95 ^ab^	89.65 ± 3.31 ^ab^	90.13 ± 7.31 ^ab^	83.79 ± 3.63 ^a^
	RTS	97.22 ± 7.62 ^bc^	89.79 ± 6.04 ^ab^	98.74 ± 5.07 ^bc^	94.16 ± 12.53 ^bc^	100.71 ± 9.98 ^c^	90.69 ± 6.40 ^bc^	94.58 ± 6.10 ^bc^	80.67 ± 4.21 ^a^
	WTW	133.08 ± 10.92 ^c^	125.84 ± 6.64 ^c^	113.68 ± 7.25 ^b^	108.39 ± 6.84 ^b^	104.99 ± 4.91 ^ab^	109.16 ± 4.29 ^b^	95.83 ± 5.37 ^a^	80.10 ± 4.51 ^a^
	RTW	132.03 ± 10.75 ^d^	116.43 ± 1.59 ^c^	111.68 ± 8.00 ^bc^	104.18 ± 0.87 ^b^	111.74 ± 5.84 ^bc^	101.23 ± 4.14 ^b^	76.28 ± 2.71 ^a^	77.50 ± 3.39 ^a^
Blue (B)	WTS	87.63 ± 5.70 ^cd^	90.13 ± 2.59 ^d^	87.14 ± 6.59 ^cd^	82.21 ± 5.93 ^bc^	77.58 ± 5.47 ^ab^	77.91 ± 2.27 ^ab^	78.37 ± 4.72 ^ab^	73.26 ± 2.39 ^a^
	RTS	87.63 ± 5.70 ^d^	80.51 ± 4.38 ^b^	86.47 ± 3.53 ^bc^	83.02 ± 8.46 ^bc^	85.81 ± 6.51 ^bc^	80.42 ± 4.43 ^b^	80.98 ± 3.34 ^bc^	72.02 ± 2.06 ^a^
	WTW	114.98 ± 9.87 ^c^	113.60 ± 5.86 ^c^	103.10 ± 8.43 ^b^	98.87 ± 3.93 ^b^	93.30 ± 4.22 ^b^	94.06 ± 3.16 ^b^	78.93 ± 4.07 ^a^	64.11 ± 3.57 ^a^
	RTW	113.41 ± 9.04 ^d^	106.02 ± 1.50 ^cd^	99.78 ± 6.91 ^bc^	94.38 ± 2.60 ^b^	100.07 ± 6.35 ^bc^	83.83 ± 3.37 ^a^	94.13 ± 2.80 ^b^	62.40 ± 2.93

* WTS, thawing by water and sample slicing; WTW, thawing by water and whole sample; RTS, thawing at room temperature and sample slicing; RTW, thawing at room temperature and whole sample. ** Means with different superscripts within each row indicate significant differences by Duncan’s multiple range test (*p* < 0.05).

**Table 4:** Model performance and classification accuracy for differentiating thawing methods using PLS-DA.

Storage Days	Model Performance * (Train)			Classification Accuracy (Test)	
	*n*	R_C_^2^	RMSEC	*n*	Accuracy (%)
0	70	0.6734	0.4813	30	80.00
1	70	0.9547	0.1064	30	100.00
2	70	0.7683	0.2407	30	96.67
3	70	0.8168	0.2140	30	100.00

* *n*, the number of samples; R_C_^2^, coefficient of determination of calibration; RMSEC, root mean square error of calibration.

## Data Availability

The original contributions presented in this study are included in the article. Further inquiries can be directed to the corresponding author.
